# Long-term impact of a community-led sanitation campaign in India, 2005–2016

**DOI:** 10.2471/BLT.18.221572

**Published:** 2019-05-28

**Authors:** Jennifer Orgill-Meyer, Subhrendu K Pattanayak, Namrata Chindarkar, Katherine L Dickinson, Upendra Panda, Shailesh Rai, Barendra Sahoo, Ashok Singha, Marc Jeuland

**Affiliations:** a Department of Government and Public Health Program, Franklin and Marshall College, Lancaster, PA, United States of America (USA).; b Sanford School of Public Policy, Duke University, Durham, USA.; c Lee Kuan Yew School of Public Policy, National University of Singapore, Singapore.; d Colorado School of Public Health, Aurora, USA.; e CTRAN Consulting, Bhubaneswar, India.; f Amnesty International, Karnataka, India.

## Abstract

**Objective:**

To evaluate the long-term impact of a community-led total sanitation campaign in rural India.

**Methods:**

Local organizations in Odisha state, India worked with researchers to evaluate a community-led total sanitation campaign, which aimed to increase the demand for household latrines by raising awareness of the social costs of poor sanitation. The intervention ran from February to March 2006 in 20 randomly-selected villages and 20 control villages. Within sampled villages, we surveyed a random subset of households (around 28 households per village) at baseline in 2005 and over the subsequent 10-year period. We analysed changes in latrine ownership, latrine functionality and open defecation among approximately 1000 households. We estimated linear probability models that examined differences between households in intervention and control villages in 2006, 2010 and 2016.

**Findings:**

In 2010, 4 years after the intervention, ownership of latrines was significantly higher (29.3 percentage points; 95% confidence interval, CI: 17.5 to 41.2) and open defecation was significantly lower (−6.8 percentage points; 95% CI: −13.1 to −1.0) among households in intervention villages, relative to controls. In 2016, intervention households continued to have higher rates of ever owning a latrine (26.3 percentage points; 95% CI: 20.9 to 31.8). However, latrine functionality and open defecation were no longer different across groups, due to both acquisition of latrines by control households and abandonment and deterioration of latrines in intervention homes.

**Conclusion:**

Future research should investigate how to maintain and rehabilitate latrines and how to sustain long-term behaviour change.

## Résumé

**Objectif:**

Évaluer l'impact à long terme d'une campagne d'assainissement total pilotée par la collectivité dans l'Inde rurale.

**Méthodes:**

Des organisations locales de l'État d'Odisha, en Inde, ont travaillé avec des chercheurs afin d'évaluer une campagne d'assainissement total pilotée par la collectivité, qui visait à accroître la demande de latrines domestiques en sensibilisant aux coûts sociaux d'un mauvais assainissement. L'intervention s'est déroulée de février à mars 2006 dans 20 villages sélectionnés aléatoirement et 20 villages témoins. Dans les villages en question, nous avons étudié un sous-ensemble aléatoire de foyers (environ 28 foyers par village) au point de référence en 2005 et sur les 10 années suivantes. Nous avons analysé les changements en matière de possession de latrines, de fonctionnalité des latrines et de défécation en plein air pour environ 1000 foyers. Nous avons estimé des modèles de probabilité linéaire examinant les différences entre les foyers bénéficiaires de l'intervention et les villages témoins en 2006, 2010 et 2016.

**Résultats:**

En 2010, 4 ans après l'intervention, la possession de latrines était nettement plus élevée (29,3 points de pourcentage; intervalle de confiance de 95%, IC: 17,5 à 41,2) et la défécation en plein air nettement plus faible (−6,8 points de pourcentage; IC 95%: −13,1 à −1,0) dans les foyers des villages ayant bénéficié de l'intervention que dans les villages témoins. En 2016, les foyers bénéficiaires de l'intervention continuaient d'avoir un taux de possession de latrines supérieur (26,3 points de pourcentage; IC 95%: 20,9 à 31,8). Cependant, les groupes ne présentaient plus de différences quant à la fonctionnalité des latrines et à la défécation en plein air, en raison de l'acquisition de latrines par les foyers témoins et de l'abandon et de la détérioration des latrines dans les foyers qui avaient bénéficié de l'intervention.

**Conclusion:**

Les recherches futures devraient s'intéresser aux moyens d'entretenir et de rénover les latrines et de faire changer durablement les comportements.****

## Resumen

**Objetivo:**

Evaluar el impacto a largo plazo de una campaña de saneamiento total liderada por la comunidad en las zonas rurales de la India.

**Métodos:**

Organizaciones locales en el estado de Odisha, India, trabajaron con investigadores para evaluar una campaña de saneamiento total liderada por la comunidad, cuyo objetivo era aumentar la demanda de letrinas domésticas mediante la concienciación sobre los costes sociales de un saneamiento deficiente. La intervención se llevó a cabo entre febrero y marzo de 2006 en 20 aldeas seleccionadas al azar y 20 aldeas de control. Dentro de las aldeas muestreadas, se encuestó a un subconjunto aleatorio de hogares (alrededor de 28 hogares por aldea) al inicio del estudio en 2005 y durante el período de 10 años subsiguiente. Se analizaron los cambios en la propiedad de las letrinas, la funcionalidad de estas y la defecación al aire libre en aproximadamente 1 000 hogares. Se estimaron modelos de probabilidad lineal que examinaron las diferencias entre los hogares de las aldeas de intervención y control en 2006, 2010 y 2016.

**Resultados:**

En 2010, cuatro años después de la intervención, la propiedad de las letrinas fue significativamente mayor (29,3 puntos porcentuales; intervalo de confianza del 95 %: 17,5 a 41,2) y la defecación al aire libre fue significativamente menor (6,8 puntos porcentuales; IC del 95 %: 13,1 a 1,0) en los hogares de las aldeas de intervención, en relación con los controles. En 2016, los hogares de intervención continuaron teniendo tasas más altas de poseer una letrina (26,3 puntos porcentuales; IC del 95 %: 20,9 a 31,8). Sin embargo, la funcionalidad de las letrinas y la defecación al aire libre ya no eran diferentes entre los grupos, debido tanto a la adquisición de letrinas por los hogares de control como al abandono y deterioro de las letrinas en los hogares de intervención.

**Conclusión:**

Los estudios futuros deberían investigar cómo mantener y rehabilitar las letrinas y cómo mantener un cambio de comportamiento a largo plazo.

## ملخص

**الغرض:**

تقييم التأثير طويل الأجل لحملة الصرف الصحي الشاملة بقيادة مجتمعية في الريف الهندي.

**الطريقة:**

تعاونت المنظمات المحلية في ولاية أوديشا بالهند مع الباحثين بهدف تقييم حملة الصرف الصحي الشاملة بقيادة المجتمع، والتي تهدف إلى زيادة الطلب على المراحيض المنزلية من خلال زيادة الوعي بالتكاليف الاجتماعية لسوء الصرف الصحي. استمر التدخل من فبراير/شباط إلى مارس/آذار 2006 في 20 قرية تم اختيارها بشكل عشوائي و20 قرية تحت المراقبة. قمنا بمسح مجموعة فرعية عشوائية من الأسر في القرى المختارة (حوالي 28 أسرة لكل قرية) في الأساس في عام 2005، وخلال فترة 10 سنوات لاحقة. قمنا بتحليل التغييرات في ملكية المراحيض، وتشغيل المراحيض، والتغوط في الأماكن المفتوحة بين قرابة 1000 أسرة. قمنا بتقدير نماذج الاحتمالات الخطية التي ركزت على الاختلافات بين الأسر في قرى الخاضعة للتدخل والمراقبة في أعوام 2006 و2010 و2016.

**النتائج:**

في عام 2010، بعد 4 سنوات من التدخل، كانت ملكية المراحيض أعلى بشكل ملموس (29.3 نقطة مئوية؛ فاصل الثقة 95%: 17.5 إلى 41.2) كما أصبح التغوط في الأماكن المفتوحة أقل بشكل ملحوظ (-6.8 نقطة مئوية؛ فاصل الثقة 95%: -13.1 إلى -1.0) بين القرى الخاضعة للتدخل، بالنسبة إلى القرى الأخرى الخاضعة للتحكم. في عام 2016، استمرت الأسر الخاضعة للتدخل في تحقيق معدلات أعلى في امتلاك المراحيض (26.3 نقطة مئوية؛ فاصل الثقة 95%: 20.9 إلى 31.8). ومع ذلك، لم يعد تشغيل المراحيض والتغوط في الأماكن المفتوحة مختلفاً عبر المجموعات، وذلك بسبب سعي الأسر الخاضعة للتحكم لامتلاك المراحيض، وكذلك التخلي عن المراحيض وتدهورها لدى الأسر الخاضعة للتدخل.

**الاستنتاج:**

يجب أن تقوم الأبحاث المستقبلية بتقصي كيفية الحفاظ على المراحيض وإعادة تأهيلها، وكذلك كيفية الحفاظ على التغيير الطويل الأجل في السلوك. ****

## 摘要

**目的:**

评估印度农村地区社区主导型“全民卫生运动”的长期影响。

**方法:**

印度奥里萨邦的当地组织与研究人员共同合作评估了一项社区主导型“全民卫生运动”，旨在提高人们对卫生条件差所带来的社会成本的意识，从而提升对家用厕所的需求。从 2006 年 2 月至 3 月，对 20 个随机挑选的村庄和另外 20 个村庄（对照组）采取干预。在抽样村庄中，我们以 2005 年和随后 10 年为基线，随机调查了一个家庭子集（每个村庄约有 28 户）。我们分析了约 1000 户家庭的厕所拥有率、厕所功能和露天排便等方面的变化。我们采用线性概率模型对被干预村庄和对照组村庄的家庭在 2006、2010 和 2016 的数据进行估算得出差异。

**结果:**

2010 年，历经 4 年的干预，相较于对照组，被干预村庄的家庭的厕所拥有率显著提高（29.3 个百分点；95% 置信区间，CI：17.5 至 41.2），露天排便显著降低（–6.8 个百分点；95% 置信区间：–13.1 至–1.0）。2016 年，被干预村庄的家庭拥有厕所的比率持续上升（26.3 个百分点；95% 置信区间：20.9 至 31.8）。然而，由于对照组中的家庭也修建了厕所，以及被干预村庄的家庭中的厕所废弃和坍坏，不同组之间的厕所功能和露天排便数据不再有差异。

**结论:**

未来研究应探讨如何维修和翻新厕所，以及如何保持长期的行为改变。

## Резюме

**Цель:**

Оценить долгосрочное воздействие возглавляемой общественностью компании за полную санитарию в сельских регионах Индии.

**Методы:**

Местные организации в штате Одиша, Индия, работали с исследователями для оценки проводимой общественностью кампании за полную санитарию, задачей которой было повышение спроса на домашние уборные путем повышения осведомленности о последствиях плохой санитарии для общества. Вмешательство длилось с февраля по март 2006 года в 20 случайным образом выбранных деревнях и в 20 контрольных деревнях. В пределах выборки авторы обследовали случайную подгруппу семейств (около 28 в каждой деревне), базовое состояние фиксировалось на 2005 год, а затем велось наблюдение на протяжении 10 лет. Были проанализированы изменения во владении уборными, их функционировании и в частоте случаев дефекации на открытом воздухе. Всего было охвачено около 1000 семейств. Были составлены приблизительные линейные модели для оценки разницы между семействами, проживающими в охваченных вмешательствами и контрольных деревнях в 2006, 2010 и 2016 годах.

**Результаты:**

В 2010 году, спустя 4 года после начала вмешательства, уровень владения уборными существенно повысился (29,3 процентного пункта; 95%-й ДИ: 17,5–41,2), а случаи дефекации на открытом воздухе значительно уменьшились (− 6,8 процентного пункта; 95%-й ДИ: от −13,1 до −1,0) среди семейств, проживающих в охваченных вмешательством населенных пунктах в сравнении с контрольными. В 2016 году семейства, проживающие в районе осуществления вмешательства, по-прежнему имели высокий уровень владения уборными (26,3 процентного пункта; 95%-й ДИ: 20,9–31,8). Однако между двумя группами уже не было разницы в функционировании уборных и уровне дефекации на открытом воздухе из-за покупки уборных контрольными семействами и из-за того, что в домах, охваченных вмешательством, они приходили в негодность.

**Вывод:**

В дальнейшем исследователям предстоит выяснить, как следует обслуживать и восстанавливать уборные и как добиться долговременного устойчивого изменения поведения.

## Introduction

Over 800 million people defecate in the open rather than use a toilet.^1^ Though global mortality from diarrhoeal diseases has recently declined,^1^ poor water and sanitation are responsible for hundreds of thousands of child deaths, as well as high rates of diarrhoea, malnutrition and stunting.^2^^,^^3^ Given the strong link between health, human development and productivity,^4^ and the benefits of privacy, dignity and gender empowerment,^5^ improving sanitation remains a key sustainable development goal.^6^ Crucial in achieving objectives for improved sanitation is the need for long-term changes in behaviour that are deeply embedded in community cultures and norms.^7^ Weak institutions, insufficient local capacity and dysfunctional markets for sanitation improvements make it especially challenging to achieve sustained behaviour change.^8^

At least two major knowledge gaps have hampered prior efforts to overcome barriers to better sanitation. First, scaling-up sanitation initiatives requires translating successful trials of their efficacy to achieve widespread use.^9^^,^^10^ For example, community-led approaches recognize that effective promotion must change deep-seated attitudes and practices, rather than simply providing a latrine. Despite the appeal of community-led total sanitation, rigorous tests of its implementation are rare, and questions remain concerning the value of various promotion strategies. A second major gap is that few studies in the water, sanitation and hygiene sector have examined whether behaviour changes following an intervention are sustained in the long term. Several studies have rigorously evaluated the short-term behavioural and health impacts of sanitation interventions.^11^^–^^16^ Yet recent systematic reviews^17^^–^^21^ identified only one peer-reviewed sanitation evaluation with a control group that examined the impact beyond 3 years. A study in 1996 showed that while diarrhoea rates were lower 6 years after a water, sanitation and hygiene intervention, only 63.9% of the latrines (425 out of 665) remained functional.^22^

A greater focus on sustainability is needed for several reasons. First, the behaviour change effects of health interventions have often been shown to decline over time;^17^ short-term evidence may therefore overestimate the true potential of interventions. Even households with access to improved sanitation may continue or revert to open defecation as time progresses.^23^ Second, deterioration of latrines due to the cost of maintenance may outweigh the benefits over time.^24^ Third, more effective programme design relies on understanding how policies improve sustainability of health interventions.

To begin to address these knowledge gaps, our study describes the evolution of latrine ownership, functionality, use and sanitation beliefs over 10 years following a community-led total sanitation intervention in Odisha state, India. We focused on these outcomes because they are key indicators of sustainability for this intervention. The study setting is particularly relevant for examining the long-term progression of sanitation behaviour; India has high rates of poor sanitation and roughly half of 1.2 billion Indians still practise open defecation.^25^ The design of the Odisha intervention drew on insights from multiple domains, and was successful in addressing short-term demand and supply barriers to behaviour change, especially among the rural poor.^11^

Our research also has policy relevance. In 2000, the government of India launched a total sanitation campaign aiming to achieve universal rural sanitation coverage by 2012, with the emphasis on community-level information, education and communication and subsidies for latrine building. However, the programme had minimal impacts on open defecation behaviour and no effect on child health outcomes.^15^ In 2014, the Swachh Bharat Mission was launched with renewed efforts to end open defecation by 2019. Despite the success of that programme in delivering millions of latrines, the persistence of open defecation^7^ highlights the need for more research that is focused on the barriers to long-term latrine use and related outcomes.^26^

The aim of our study was to examine the long-term impacts of a community-led total sanitation campaign in Odisha, India on latrine adoption and open defecation, and to study barriers to continued latrine use.

## Methods

### Baseline study

The original cluster-randomized control trial tested the effects of a community-led total sanitation-type campaign in Odisha state in 2005–2006.^11^ The study included 40 villages from two adjacent blocks in Bhadrak district (population around 1.5 million), where very little sanitation promotion had previously occurred. Twenty of these villages were randomized to receive a village-level intensive promotion campaign, while the other 20 villages served as controls. To obtain baseline data from sample villages, a random subset of households (around 28 households per village) were surveyed in August and September 2005. The baseline sample in each arm was generally representative of rural conditions in the region (Table 1; available at: http://www.who.int/bulletin/volumes/96/8/18-221572.). At the start of the study, 58.4% (630/1086) of households were below the poverty line of 368 Indian rupees per head per month. There was limited baseline sanitation among sample households (91.2%, 988/1086, reported open defecation), despite widespread dissatisfaction with village cleanliness and women’s concerns about the safety and privacy of open defecation.

**Table 1:** Baseline household characteristics and attitudes in the study of long-term impacts of a community-led total sanitation campaign in Odisha, India, 2005

Variable	No. (%) of households			*P*
	Intervention villages (*n* = 534)	Control villages (*n* = 552)	Total (*n* = 1086)	
**Household characteristics**				
Female respondent	498 (93.3)	518 (93.8)	1016 (93.6)	0.81
Below poverty line	300 (56.2)	334 (60.5)	634 (58.4)	0.38
Experienced a serious crisis or burden within the last year	241 (45.1)	288 (52.2)	529 (48.7)	0.30
Can easily obtain loan	252 (47.2)	289 (52.4)	541 (49.8)	0.36
Own a mosquito net	437 (82.1)	487 (88.2)	924 (85.2)	0.26
Own a mobile phone	5 (1.0)	13 (2.4)	18 (1.7)	0.10
Own a television	47 (8.8)	93 (16.8)	140 (12.9)	0.05
Own a bicycle	277 (54.3)	310 (58.2)	587 (56.3)	0.47
**Household water and sanitation behaviour**				
Uses an improved water source	200 (37.5)	231 (41.8)	431 (39.7)	0.60
Uses multiple water sources	357 (66.9)	319 (57.8)	676 (62.2)	0.31
Treats drinking water	50 (9.4)	72 (13.0)	122 (11.2)	0.21
Uses a private latrine for defecation	32 (6.0)	71 (12.9)	103 (9.5)	0.06
Practices open defecation	506 (94.8)	484 (87.7)	990 (91.2)	0.02
In past 24 hours, washed hands before eating	480 (89.9)	476 (86.2)	956 (88.0)	0.35
In past 24 hours, washed hands after handling child’s faeces	137 (25.7)	125 (22.6)	262 (24.1)	0.57
In past 24 hours, washed hands after defaecating	310 (58.1)	316 (57.2)	626 (57.6)	0.89
**Household attitudes**				
Believes that their village is very dirty	236 (44.4)	198 (36.0)	434 (40.1)	0.13
Completely dissatisfied with current sanitation situation	386 (72.6)	337 (61.1)	723 (66.7)	0.01
Believes that open defecation causes diarrhoea	490 (91.8)	505 (91.5)	995 (91.6)	0.92
Believes that sanitation and hygiene are the most important improvements needed in the village	66 (12.4)	110 (19.9)	176 (16.2)	0.13
Believes that women lack privacy during open defecation	172 (34.7)	164 (34.4)	336 (34.5)	0.95
Believes that women are not safe defaecating in the open at night	110 (30.8)	153 (33.8)	314 (32.3)	0.42

Notes: Twenty villages in Bhadrak district were randomized to receive a village-level intensive promotion campaign, while the other 20 villages served as controls. *P*-values for the differences are adjusted for intra-cluster correlation at the village level. *n* is the total number of households interviewed.

Source: Adapted from Pattanayak et al., 2009.^11^

From February to March 2006, a specialized field team worked closely with community-based organizations and state and local government leaders to mobilize the target population and implement the campaign in the 20 intervention villages. The campaign included community education activities on the benefits of latrines, including health, safety and dignity, and privacy for women; providing local support for building pit latrines; and awareness-raising about government subsidies for latrine building (Box 1). A post-intervention survey conducted in August and September 2006, 6 months after the intervention, showed that latrine ownership had increased by 24.1% (from 40/534 to 168/521 households) in treatment villages relative to controls (from 71/552 to 70/529 households), and had increased more among below-the-poverty-line households that were eligible for government subsidies.^11^ Additionally, the intervention was linked to improvements in child health.^27^

Box 1 **The community-led total sanitation campaign in Odisha, India, 2006**

A specialized field team worked closely with community-based organizations and state and local government leaders to implement the different components of the campaign:^11^

(i) A set of intensive information, education and communication activities that gathered villages together to evoke a collective response to widespread open defecation. Activities included:

- defecation mapping: villagers drew maps of their village and noted the relative distances between common defecation sites and water sources, crops and schools;
- walk of shame: villagers walked around their village identifying common defecation sites and noting the proximity of sites to other important community locations (e.g. water sources or health clinics); and
- faecal mass calculations: a facilitator worked with villagers to collect and weigh the faeces gathered from these village defecation sites.

(ii) Establishing accessible latrine production centres in villages. The aim was to provide materials and support for to households and reduce the costs of pit latrine construction.

(iii) Raising of people’s awareness about subsidies for latrine building. At the time, few households in the study site were taking advantage of the available subsidies from the Indian government to households below the poverty line.

The information campaign took place in 2006 only. Although village production centres created an infrastructure that enabled continuing support to households, due to limited follow-up, it is unclear whether these supply-side activities continued. Finally, while the intervention only delivered information about the availability of subsidies in 2006, the government of India continues to offer subsidies to below-the-poverty-line households.

### Follow-up study

**Fig. 1:**
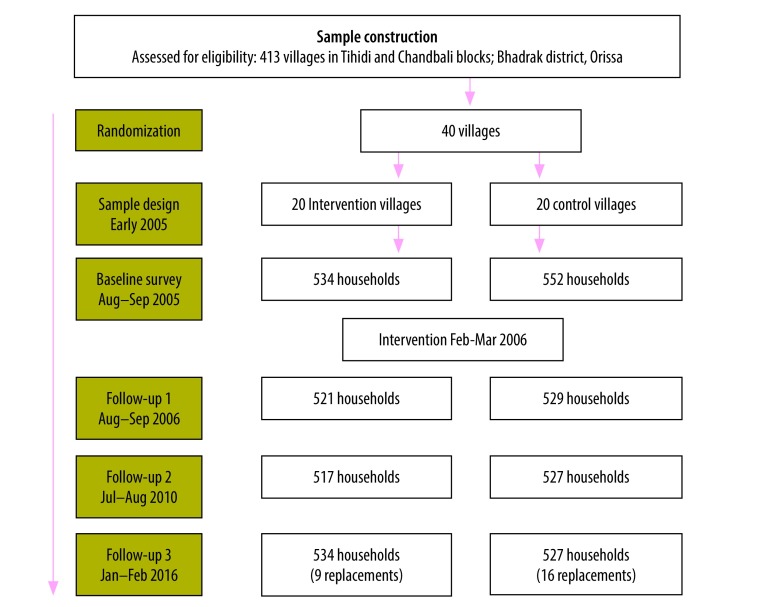
Study timeline and sampling in the study of long-term impacts of a community-led total sanitation campaign in Odisha, India, 2005–2016

Note: Selection criteria for eligible villages were: (i) medium-sized, 70–500 households per village; (ii) low risk of spillovers, one village per *panchayat*; villages not adjacent.

We examined the sustainability of changes in latrine ownership, functionality, open defecation and sanitation-related perceptions, using surveys administered to the original households about 4 and 10 years after the intervention, in July and August 2010 and January and February 2016, respectively. Surveys were conducted in Oriya by trained enumerators who were recent or current university students, although not the same as those at baseline. Additional questions were included in the later rounds to better understand latrine use and abandonment. Fig. 1 depicts the study timeline. We were able to re-survey most of the original households in 2010 (96.0%; 1043/1086 households) and 2016 (97.5%; 1059/1086 households). For households that we did not relocate in 2016, we replaced the household with a neighbouring household.

The 2010 and 2016 surveys were conducted with the approval of the Duke University intuitional review board (protocols 2799 and D0352). Local permission to conduct these surveys was obtained from village leaders and via informed consent from all participating households.

### Data analysis

To quantify the effects of the promotion effort, we used linear probability models to examine the differences between households in intervention villages and controls in 2006, 2010 and 2016. We used the following equation:
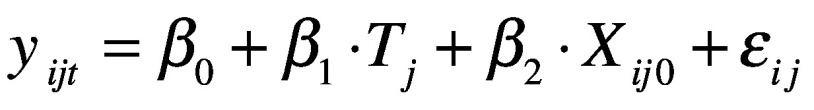
(1)where *y_ijt_* is the outcome *y* measured for household *i* in village *j* in year *t*, and *T_j_* is the treatment status of village *j.* The outcome variables that we measured were: (i) households ever owning and abandoning latrines since 2005; (ii) open defecation practices (a household was defined as practising open defecation if at least one member reports regular open defecation); (iii) variables related to latrine function, quality and investment (self-reported functionality and cost incurred for construction; observations of structural integrity; and observed problems with the slab, walls or roof); (iv) subjective perceptions of open defecation and sanitation, and aspirations for reinvestment in latrines; and (v) recall of recent sanitation promotion activities occurring in the village.

We ran separate regressions for the outcomes in 2006, 2010 and 2016. Thus, *β_1_*represents the average difference between households in intervention and control villages in a given year. To allow for intra-village correlation, we clustered standard errors *ε_ij_* for all analyses at the village level. We also controlled for the few baseline variables *X_ij0_* (i.e. baseline latrine use, television ownership and satisfaction with village sanitation) that were somewhat unbalanced between treatment and control villages in 2005. We also conducted logistic regressions and found that the results were consistent across both sets of models.

For the outcomes in categories (i), (ii) and (iv) above, we conducted the analyses using the full sample of households. In these cases, the coefficient *β_1_*represents the effect of being in an intervention village in 2005 on the outcome of interest. For category (iii), the regressions were only for households owning latrines at the time of the survey. *β_1_* in this case measures how latrine function and quality indicators vary across households owning latrines in the intervention versus control villages. For ownership and abandonment, we conducted separate analyses for the households below and above the poverty line, to assess how subsidy eligibility related to sustainability.

If nongovernmental organizations (NGOs) or local governments had become more involved in control villages after the intervention concluded, we might observe differential rates of latrine adoption and open defecation behaviour in the long term. We tested category (v) above in two ways. First, we used community surveys conducted with village leaders in 2016 that asked about the sanitation activities of NGOs and local government within the village. Second, we used the household surveys from 2010 and 2016 that asked household members to recall specific sanitation activities occurring in their village.

## Results

### Latrine adoption and ownership

**Fig. 2:**
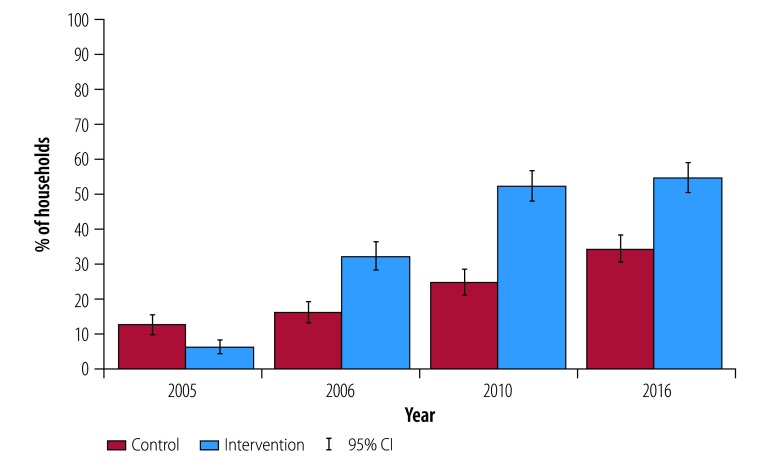
Households ever owning a latrine among study households in Odisha, India, 2005–2016

In intervention villages the proportion of households that had ever acquired a latrine was higher in the 2006 and 2010 surveys compared with 2005, with little further increase in 2016 (Fig. 2). By 2010, roughly half of in community-led total sanitation villages (271/517 households) had ever built a latrine, compared with only a quarter (131/527 households) in control villages. Controlling for baseline household variables, the increase in the percentage of households having a latrine in 2010 was 29.3 percentage points (95% confidence interval, CI: 17.5 to 41.2) higher than in control villages (Table 2). The increase was higher among below-the-poverty-line households (38.1 percentage points; 95% CI: 24.9 to 51.2), who could obtain subsidies to help defray the costs of constructing the latrine.

CI: confidence interval.

Notes: A community-led total sanitation campaign ran from February to March 2006. Numbers of households interviewed in control and intervention villages were 552 and 534 at baseline in 2005; 529 and 521 in 2006; 527 and 517 in 2010; and 534 (nine replacement households) and 552 (16 replacement households) in 2016, respectively.

**Table 2:** Current latrine ownership and abandonment and practice of open defecation among control and intervention households in the study of long-term impacts of a community-led total sanitation campaign in Odisha, India, 2005–2016

Variable		Baseline survey, 2005				Follow-up surveys										
						2006				2010				2016		
		Total no. of households	Control, mean %	Treatment effect, % points (95% CI)		Total no. of households	Control, mean %	Treatment effect, % points (95% CI)		Total no. of households	Control, mean %	Treatment effect, % points (95% CI)		Total no. of households	Control, mean %	Treatment effect, % points (95% CI)
	**Own a latrine**															
Total sample		1086	12.9	−6.5 (−13.7 to 0.7)		1048	13.2	24.1 (10.9 to 37.3)		1043	19.7	29.3 (17.5 to 41.2)		1059	27.5	1.3 (−11.2 to 13.7)
Below poverty line		634	12.0	−6.6 (−13.8 to 0.5)		608	12.5	24.7 (10.6 to 38.8)		599	19.1	38.1 (24.9 to 51.2)		515	21.7	4.6 (−7.3 to 16.6)
Not below poverty line^a^		452	14.2	−6.5 (−15.1 to 2.1)		440	14.3	23.8 (9.2 to 38.3)		444	20.5	17.1 (6.2 to 28.1)		544	32.6	−1.8 (−16.6 to 13.0)
	**Ever abandoned a latrine**															
Total sample		NA	NA	NA		1048	2.3	−0.5 (−1.9 to 0.8)		1043	4.2	4.4 (−0.8 to 9.6)		1059	3.8	21.7 (14.6 to 28.7)
Below poverty line		NA	NA	NA		608	1.9	−0.8 (−1.5 to 3.1)		599	4.4	4.4 (−2.6 to 11.4)		515	4.3	24.8 (15.3 to 34.3)
Not below poverty line^a^		NA	NA	NA		440	2.9	−2.3 (−3.8 to −0.8)		444	3.9	4.5 (−1.5 to 10.5)		544	3.4	18.2 (11.7 to 24.8)
	**Practise open defecation^b^**															
Total sample		1086	87.7	7.1 (3.7 to 10.4)		1048	90.5	−23.1 (−34.9 to −11.4)		1043	91.1	−6.8 (−13.1 to −1.0)		1059	81.7	0.6 (−9.2 to 10.4)
Below poverty line		634	89.5	7.1 (3.2 to 11.1)		608	92.2	−24.2 (−37.7 to −10.8)		599	91.6	−8 (−16.1 to −0.0)		515	85.8	−0.7 (−10.1 to 8.6)
Not below poverty line^a^		452	84.9	7.4 (1.6 to 13.3)		440	88.1	−21.7 (−34.0 to −9.4)		444	90.4	−5.1 (−11.8 to 1.7)		544	78.2	1.9 (−9.8 to 13.5)

CI: confidence interval; NA: not applicable (not observed).

^a^ Some households did not report their poverty-line status and were included as not below the poverty line.

^b^ Open defecation here refers to a household having at least one member practising open defecation on a regular basis.

Notes: The intervention ran from February to March 2006. We collected baseline data from household samples in 20 intervention villages and 20 control villages in August and September 2005. Follow-up surveys in mostly the same households were carried out in August and September 2006, July and August 2010, and January and February 2016. These estimates of outcomes are from linear probability models. The 2006, 2010 and 2016 models include control variables that were unbalanced at baseline, and do not include replacement households (added in 2016). Treatment effect (%) reports the coefficient on the treatment variable from these linear probability models, thus measuring the difference between the means in treatment and control villages. Results omitting controls, available on request from the corresponding author, were not substantively different. Standard errors are clustered at the village level.

**Fig. 3:**
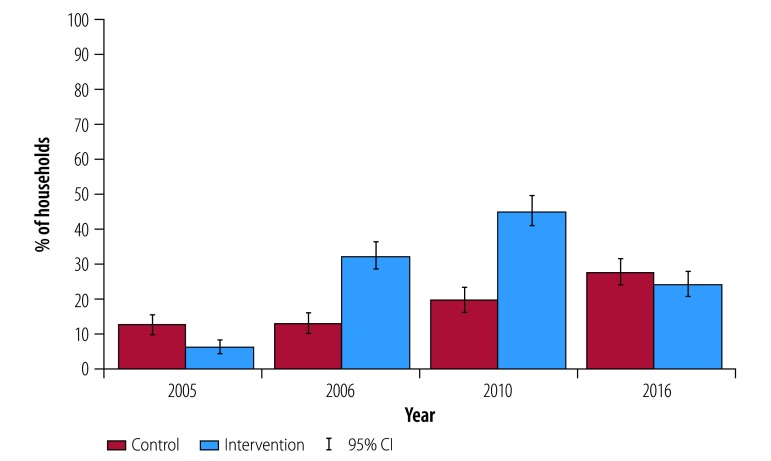
Households currently owning a latrine among study households in Odisha, India, 2005–2016

Despite the fact that intervention households were more likely to have ever owned a latrine in 2016 (Fig. 2), there was no significant difference in current ownership of a latrine between treatment and control villages in 2016 (Fig. 3), due to higher rates of adoption by new households in control villages after 2010 and latrine abandonment of intervention households. Nearly all intervention households who acquired latrines after 2005 were still using them in 2010, but by 2016 more households in intervention villages reported having ever abandoned a latrine (21.7 percentage points; 95% CI: 14.6 to 28.7; Table 2). Consequently, there were no significant differences in ownership rates in 2016 between control and intervention villages (1.3 percentage points; 95% CI: −11.1 to 13.7). Correlates of latrine adoption and the effects of the intervention on latrine use across different ages and gender can be found in the data repository.^28^

CI: confidence interval.

Notes: A community-led total sanitation campaign ran from February to March 2006. Numbers of households interviewed in control and intervention villages were 552 and 534 at baseline in 2005; 529 and 521 in 2006; 527 and 517 in 2010; and 534 (nine replacement households) and 552 (16 replacement households) in 2016, respectively.

### Open defecation

**Fig. 4:**
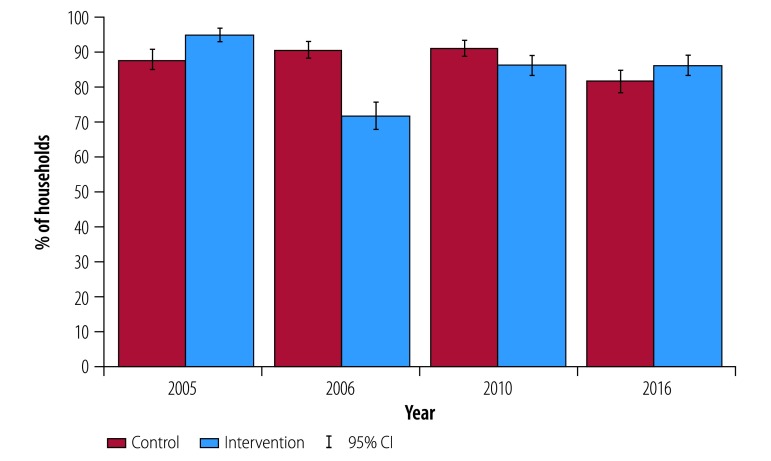
Households reporting open defecation among study households in Odisha, India, 2005–2016

Consistent with these differential patterns in ownership across villages, there was a decline in open defecation in the short-term between the 2005 and 2006 surveys among intervention villages (from 94.8% to 71.8%) relative to control villages (from 87.8% to 90.5%; Fig. 4). This small decline relative to latrine adoption suggests relatively low latrine usage. Controlling for differences in baseline variables, households in intervention communities in 2006 were less likely (−23.1 percentage points; 95% CI: −34.9 to −11.4 to practise open defecation than households in control communities (Table 2). The percentage of households practicing open defecation remained lower in intervention villages (−6.8 percentage points; 95% CI: −13.1to −1.0) compared with control villages in 2010 (Table 2). However, open defecation in intervention villages increased relative to 2006 (from 374/521 to 446/517), even as latrine ownership increased in this group (Fig. 3). Since latrine abandonment was relatively low in 2010, these higher rates of open defecation suggest that households did not fully switch to using latrines 4 years after the intervention. In the longer-term (2016), open defecation rates in intervention and control villages converged (460/534 versus 451/552; *P* = 0.90), again due to both a decline in open defecation in control villages (Fig. 4) and abandonment of latrines in intervention villages (Fig. 3). The effect of the intervention on open defecation by individuals of different ages and sexes can be found in the data repository.^28^

CI: confidence interval.

Notes: A community-led total sanitation campaign ran from February to March 2006. Numbers of households interviewed in control and intervention villages were 552 and 534 at baseline in 2005; 529 and 521 in 2006; 527 and 517 in 2010; and 534 (nine replacement households) and 552 (16 replacement households) in 2016, respectively.

### Latrine abandonment

**Fig. 5:**
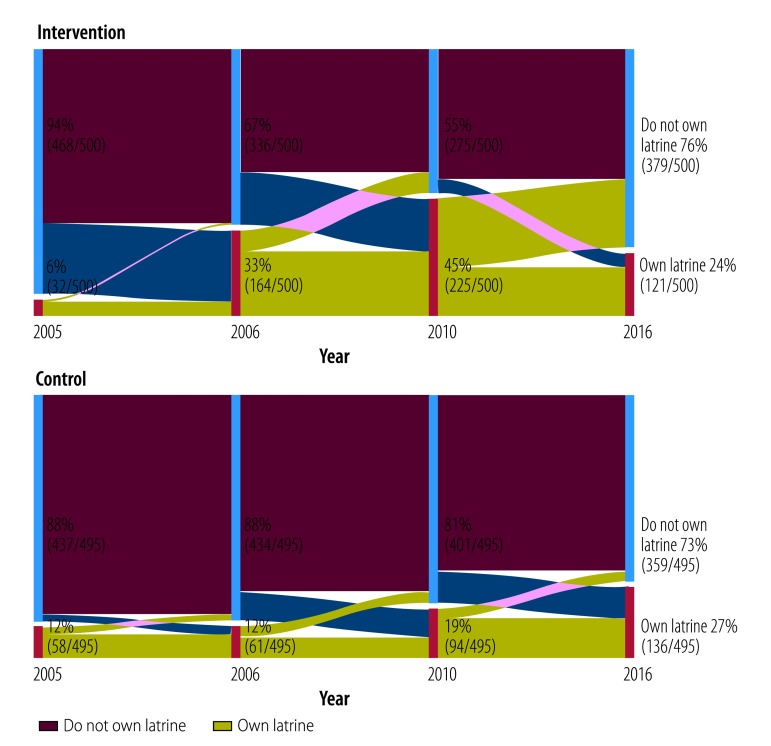
Ownership and abandonment of latrines among study households in Odisha, India, 2005–2016

Notes: A community-led total sanitation campaign ran from February to March 2006. The sample used in this figure were the households that remained in the study for the entire duration of the study (2005–2016);500 intervention households and 495 for control households. The intervention panel shows that new latrine adoption was substantial in intervention households between 2005 and 2006 and between 2006 and 2010, while between 2010 and 2016, a large proportion of intervention households abandoned latrines. Control panel shows that there were more consistent levels of latrine adoption and abandonment in control households throughout the course of the study.

Given the initial success of the intervention, and increased adoption of latrines among intervention households in 2010, it is perhaps surprising that households would abandon latrines and continue open defecation in the long term. The lack of difference in current ownership rates reported in 2016 was a combination of catch-up in control villages and abandonment in intervention villages. Fig. 3 shows that while current latrine ownership in control villages increased from 12.9% (71/552 households) to 27.5% (152/552 households) between 2005 and 2016, current latrine ownership in intervention villages decreased from 44.7% (231/517 households) to 24.2 (129/534 households) between 2010 and 2016. Latrine adoption and abandonment rates were fairly stable in control communities. However, intervention communities experienced a large increase in latrine ownership in 2006 and 2010, followed by a high rate of abandonment in 2016. Fig. 5 depicts the dynamics of latrine adoption and abandonment more clearly. While there was substantial adoption of new latrines in intervention households in 2005–2006 and up to 2010, many households abandoned latrines after 2010. In contrast, control villages showed more consistent levels of latrine adoption and abandonment throughout the period of the study. 

The latrine pit being full did not appear to be a major reason for abandonment. Few households owning latrines in 2016 reported ever needing to empty their pit (31/135 treatment households and 29/153 control households), which points to relatively low use of latrines. Our data suggest, however, that construction quality was an important factor. By 2016, latrines were more likely to have observable structural problems in intervention relative to control households (effect size: 24.1%; SE: 5.8) and existing latrines were less likely to be functional in intervention households (−26.3%; SE: 5.6; Table 3). Latrines in intervention villages were generally 2.6 years older than those in control villages (*P* = 0.02) and were more likely to have problems in 2010 and 2016, whereas in 2006 these latrines were new and less likely to have problems (Table 3).

**Table 3:** Outcomes of the community-led total sanitation-type intervention among control and intervention households in Odisha, India, 2006–2016

Variable	Follow-up surveys													
	2006					2010					2016				
	Total no. of households	Control, mean	Treatment effect (SE)	*P*		Total no. of households	Control, mean	Treatment effect (SE)	*P*		Total no. of households	Control, mean	Treatment effect (SE)	*P*	
**All households**															
Recall sanitation promotion in past 5 years, %	1050	9.6	58.1 (4.6)	< 0.01		1043	82.9	5.8 (3.7)	0.12		1059	27.2	−18.7 (6.2)	< 0.01	
Plan to build new latrine in future, %	1041	31.9	15.7 (4.8)	< 0.01		1043	34.0	1.2 (5.8)	0.84		1059	67.0	11.5 (5.0)	0.03	
Practise open defecation, %	1048	90.5	−23.0 (5.8)	< 0.01		1043	91.1	6.8 (3.1)	0.03		1059	81.7	0.6 (4.9)	0.90	
Have felt pressure from villagers to build a latrine, %	NA	NA	NA	NA		1042	22.4	11.8 (5.9)	0.05		1059	28.4	0.5 (4.0)	0.91	
**Current latrine owners only**															
Time since acquisition of first latrine, among current owners, years	153	0.4	−0.3 (0.2)	0.13		314	12	−0.3 (1.0)	0.79		348	6.7	2.6 (1.0)	0.02	
Was household’s idea to build latrine, %	156	80.0	−18.3 (14.9)	0.24		315	79.2	−21.5 (8.5)	0.02		348	79.1	−21.3 (8.3)	0.02	
Latrine is functional, among current owners, %	NA	NA	NA	NA		NA	NA	NA	NA		NA	95.5	−26.3 (5.6)	< 0.01	
Latrine has a structural problem, %	238	78.6	−32.5 (9.0)	< 0.01		315	26.7	7.6 (10.3)	0.46		348	3.8	24.1 (5.8)	< 0.01	
Latrine has a problem with walls, %	NA	NA	NA	NA		315	48.5	25.2 (9.2)	0.01		285	35.5	17.2 (9.6)	0.08	
Latrine has been emptied, %	NA	NA	NA	NA		NA	NA	NA	NA		284	10.5	8.4 (0.06)	0.19	
Reported cost of latrine, Indian rupees	96	4227	−3144.3 (2644.0)	0.25		313	6457	−1734.2 (917.2)	0.07		342	11 941.6	−4282.0 (2 499.9)	0.09	

NA: not applicable (not observed); SE: standard error.

Notes: The intervention ran from February to March 2006. We collected baseline data from household samples in 20 intervention villages and 20 control villages in August and September 2005. Follow-up surveys in mostly the same households were carried out in August and September 2006, July and August 2010, and January and February 2016. All estimates of outcomes are from linear probability models that include control variables that were unbalanced at baseline. Results omitting controls, available on request from the corresponding author, were not substantively different. All estimates pertain to the effect of the 2006 intervention, as conducted at the community level, and are thus intention-to-treat results. SE of the treatment effects are clustered at the village level. Not all outcomes were observed for all households, as indicated by the sample sizes.

Memory of the promotion effort appears to have faded quickly. In 2006 more households in intervention villages remembered sanitation promotion activities (effect size versus controls: 58.1%; SE: 4.6), whereas in 2010 there was no significant difference (effect size: 4.8%; SE: 3.7) and by 2016 households in treatment villages were less likely to remember promotion activities (effect size: −18.7%; SE: 6.2; Table 3). In addition, a high percentage of control households in 2010 reported remembering promotion activities (82.9%; 437/527) and it is therefore possible that recent sanitation activities in these villages were partially responsible for the increase in latrines in control villages. Compared with control households, more households in intervention villages reported pressure to build latrines from community members in 2010 (effect size: 11.8%; standard error of mean, SE: 5.9), but not in 2016 (effect size: −0.5%; SE: 4.0). Even so, more households in intervention villages aspired to rebuild latrines than those in control villages, even in 2016 (effect size: 12.5%; SE: 5.0; Table 3). The correlates of latrine abandonment can be found in the data repository.^28^

### Involvement of other organizations

Respondents in treatment and control villages reported similar levels of NGO involvement (effect size: 0.0%; SE: 9.7) and government sanitation assistance (effect size: 5.0%; SE: 13.5; Table 4). Households in control villages remembered sanitation activities taking place in their villages in 2010 and 2016 more than did households in treatment villages (Table 3 and Table 4). Thus, some of the increase in control latrine ownership over the course of the study was likely due to more sanitation promotion happening in control villages following the intervention. 

**Table 4:** Households’ and village leaders’ recall of community-led sanitation activities within control and intervention villages in Odisha, India, 2006–2016

Variable	2010					2016			
	No. of household or villages	Mean % among controls	Treatment effect, % points (SE)	*P*		No. of households or villages	Mean % among controls	Treatment effect, % points (SE)	*P*
**Households’ recall of activities**									
Any sanitation activities	1043	83.1	5.8 (3.7)	0.12		1059	30.2	–18.7 (6.2)	< 0.01
Walk of shame	NA	NA	NA	NA		1059	15.6	–10.3 (4.0)	0.01
Defecation mapping	NA	NA	NA	NA		1059	3.4	–3.4 (1.8)	0.06
Faecal calculations	NA	NA	NA	NA		1059	1.0	–1.0 (0.8)	0.35
Demonstration of latrine technologies	NA	NA	NA	NA		1059	24.9	–15.4 (5.4)	< 0.01
Messages on TV and radio	1043	40.1	–2.8 (6.1)	0.65		1059	93.7	–1.4 (3.1)	0.65
Posters and pamphlets	1043	6.6	2.2 (2.1)	0.31		1059	10.4	–5.5 (2.8)	0.06
**Village leaders’ recall of activities**									
NGO involvement in sanitation activities	NA	NA	NA	NA		40	10.0	0.0 (9.7)	1.00
Government assistance for sanitation activities	NA	NA	NA	NA		40	20.0	5.0 (13.5)	0.71

NA: not applicable (not observed); NGO: nongovernmental organization; SE: standard error.

Notes: All estimates are from linear probability models that include control variables that were unbalanced at baseline. The results omitting controls, available upon request from the corresponding author, are not substantively different. Treatment effect (%) reports the coefficient on the treatment variable from these linear probability models, thus measuring the difference between the means in treatment and control villages. All estimates pertain to the effect of the 2006 community led total sanitation intervention, as conducted at the community level, and are thus intention-to-treat results. In 2016, we asked respondents if they remembered any of the sanitation activities within the past 5 years. In 2010, we asked respondents if they remembered any of the sanitation activities ever occurring, with a follow-up question about the timing of the activity. SEs of the treatment effects are clustered at the village level.

## Discussion

We returned to the site of an intensive behaviour change campaign and found that acquisition and use of latrines remained higher in treatment villages 4 years after the intervention. The medium-term success of this intervention can be attributed to several features. First, the campaign focused on a range of sanitation benefits, including health, privacy and dignity, and therefore helped to motivate the use of latrines. Second, the intervention recruited local builders and created an infrastructure for continued sales of latrine materials even after the intervention ended, which allowed households to make investments even after 2006. Third, the campaign targeted communities, creating the possibility of spillovers across peers and over time. Finally, the intervention helped to connect below-the-poverty-line households with government-sponsored subsidies, rendering latrines more affordable. While we observed many new latrines in 2010 relative to 2006, those facilities could have been built long before 2010. Indeed, many treatment households reported their intention to build a latrine in 2006,^11^ likely spurred on by the behaviour change campaign implemented earlier that year. The numbers of new latrines acquired up to 2010 suggest that the promotion campaign had effects on investment that persisted for at least 4 years, even as the conventional national campaign was progressing in the background.

Despite this medium-term success, after 10 years there was no significant difference in latrine ownership and open defecation between treatment and control communities. Some of the decline in the effect of the original campaign was due to acquisition of latrines over time in control villages, which could be the result of increased sanitation activity in control villages following the intervention. Yet this decline was also driven by treatment households abandoning latrines or discontinuing use. There are several reasons why a household might abandon a latrine. Latrines might have filled up, and households lacked the know-how or financial resources to empty them; maintenance costs for latrines might have increased over time; and community-led total sanitation may not have sufficiently shifted local norms favouring open defecation.

Our results suggest three main challenges in sustaining improved sanitation behaviour, relating to both hard (infrastructure) and soft (information, education and communication) inputs. First, open defecation may persist even among households that built latrines; latrine use thus did not become a universal social norm. Second, campaign messages lose effect over time, which may contribute to the persistence of open defecation even in intervention villages. More regular messaging may be critical to overcome short memories and deep-rooted habits.^7^^,^^29^ Third, the quality of latrines deteriorates over time, creating a risk that households will abandon them. Given household budget constraints and thin supply chains for maintenance services in our study setting, ongoing financial support for latrine upkeep (parts and labour) may be necessary to sustain the impact of interventions.^30^

In the context of quasi-public goods, such as sanitation and especially in resource-poor settings with affordability challenges, continued provision of information and maintenance of the infrastructure often requires governmental or other institutional support. Such support should not be assumed, however. While government and donors continue to work to increase sanitation coverage among the poor, they typically devote less attention to maintaining investments. Our findings are particularly important in the context of the current Swachh Bharat Mission programme in India, which primarily focuses on infrastructure provision. Given the high cost of the programme, it is important that policy-makers do not neglect the elements required to ensure persistent declines in open defecation and hence longer-term benefits.

Our findings also point to the need for more work that examines the success of interventions beyond a few years. More research on the sustainability of sanitation interventions would help to confirm whether the findings of this study are relevant to experiences in other contexts. Such long-term research is uncommon in public health and other applied fields, in part because it is so costly. Nonetheless, understanding how and why programmes affect outcomes in the long term is necessary for improving the sustainability of public health interventions.
